# A Case of Rosai-Dorfman Disease Successfully Treated by Corticotherapy

**DOI:** 10.1155/2024/9965038

**Published:** 2024-04-22

**Authors:** Alioune Badara Diallo, Moustapha Ndiaye, Moussa Seck, Mohamed Keita, Elimane Seydi Bousso, Sokhna Aissatou Touré, Blaise Félix Faye, Saliou Diop

**Affiliations:** ^1^Hematology Department, Cheikh Anta Diop University, Dakar, Senegal; ^2^ENT Department, HOGIP Hospital Center, Dakar, Senegal

## Abstract

Rosai-Dorfman disease (RDD) is a benign histiocytic proliferation that results in nodal and extranodal involvements. It is a rare disease, with fewer than 1,000 cases reported in the literature, which explains its lack of knowledge by physicians and the lack of codified therapeutic strategies. We report the case of an 8-year-old girl who presented a rapidly progressive cervical lymph node mass; the diagnosis of RDD was made based on histology and immunohistochemistry. The patient was treated with oral corticosteroids at a dose of 1 mg/kg/d with a favorable outcome and no recurrence after one year of follow-up. This observation illustrates the clinical presentation and diagnosis of this rare clinicopathological entity. The prognosis and treatment options are also discussed.

## 1. Introduction

Rosai-Dorfman disease (RDD), or sinus histiocytosis with lymphadenopathy, is a benign non-Langheransian histiocytosis. It is a rare condition of unknown cause, histologically characterized by massive histiocytic infiltration of sinus lymph nodes and/or lymphatic vessels and clinically by large tumor masses, either nodal or extranodal [1]. Typically, this disease is revealed by large bilateral, noninflammatory cervical adenopathies in 90% of the cases [1, 2].

We report here the case of an 8-year-old girl who presented with a rapidly progressive cervical lymph node mass. The diagnosis of RDD was made on the basis of disorganized lymph node histology with histiocyte infiltration and images of emperipolesis. Oral corticosteroid therapy of 1 mg/kg/d of prednisone was prescribed for a total of 2 months. Evolution was marked by rapid mass involution after 1 month of treatment, allowing degression and discontinuation of corticosteroid therapy, with no recurrence at 1 year of follow-up.

This case illustrates the clinical presentation and diagnosis of this rare clinical-pathological entity. The main differential diagnoses and treatment options are also discussed.

## 2. Case Presentation

**Figure 1:**
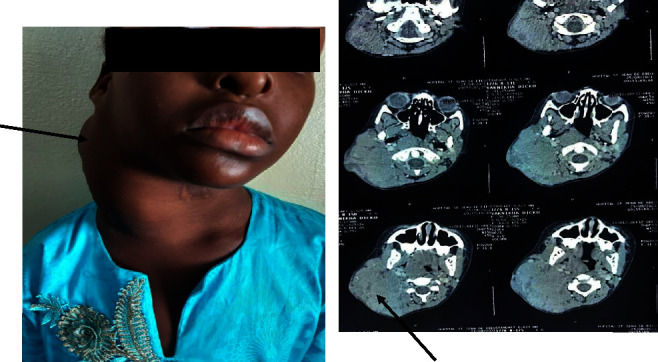
Diagnostic appearance : right laterocervical mass (black arrow) at clinical examination (a) and at CT cervical scan (b).

An 8-year-old girl, with no specific medical history, was admitted to the hospital for investigation of a cervical mass. The symptoms had been evolving for about 2 months and were marked by the appearance of a swelling in the right cervical, painless, and rapidly increasing in size. There were no compressive signs of the aerodigestive tract; in particular, there was no difficulty breathing, no dysphagia, and no change in voice timbre. There were no general signs (fever, night sweats, or weight loss). Clinical examination revealed a mass of the right laterocervical that measured 11 cm in length, was firm in consistency, and mobile in relation to the superficial planes, without the tendency to spontaneous fistulation (Figure 1). The other peripheral lymph node areas were free. There was no hepatosplenomegaly. The rest of the clinical examination was not remarkable.

The complete blood count (CBC) showed normocytic normochromic anemia, with erythrocytic anisopoikilocytosis in the blood smear, without abnormal cells circulating. The direct Coombs test was negative. Biochemical analysis revealed a biological inflammatory syndrome; the rest of serum parameters were normal. Viral serologies (HIV, HBV, and HCV) were negative. The tuberculin skin test was negative.

Cervical CT revealed clusters of right lateral cervical adenopathies with no signs of compression of the aerodigestive tract (Figure 1). The chest radiograph and abdominal ultrasound were normal.

**Figure 2:**
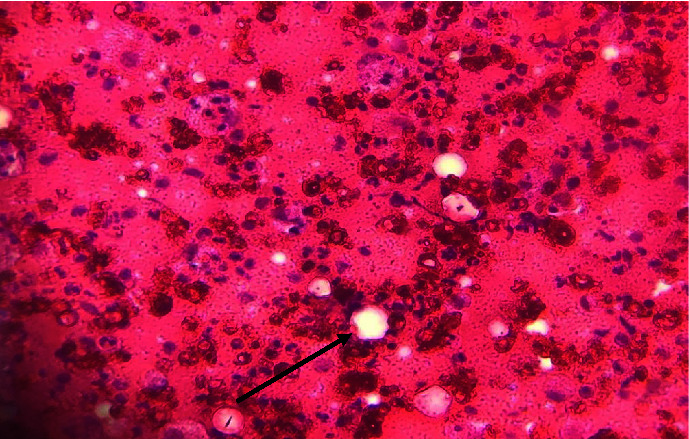
Disorganization of lymph node architecture with images of emperipolesis (black arrow).

A lymph node biopsy was then performed. Histology showed a disorganized lymph node architecture, with dilated sinuses containing numerous histiocytes that phagocytose lymphocytes and lymph node parenchyma marked by lymphocyte hyperplasia and images of emperipolesis (Figure 2). Immunohistochemistry revealed mainly histiocytic markers, in particular PS100 and CD68. The pathology favored a diagnosis of Rosai-Dorfman disease.

The patient was treated with oral corticosteroids of 1 mg/kg/d of prednisone, combined with adjuvant measures.

**Figure 3:**
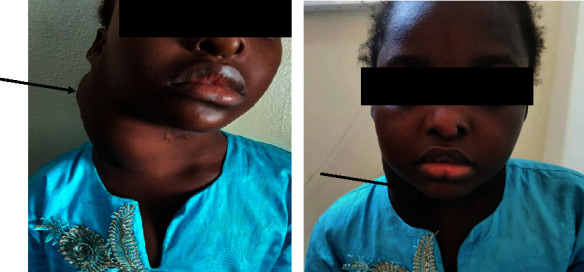
Involution of the right laterocervical mass (black arrow) after one month of corticosteroid therapy.

Evolution after 1 month of treatment was marked by involution of the cervical mass (Figure 3) and, in biological terms, normalization of the CBC and regression of the inflammatory syndrome (Table 1). The corticosteroid therapy was then reduced and stopped after a total of 2 months. Clinical monitoring revealed no recurrence after one year of follow-up.

## 3. Discussion

Rosai-Dorfman disease (RDD) was first described by Destombes in 1965. It was then individualized as a clinicopathological entity from two publications reporting 4 and then 34 observations by Foucar et al. in 1969 and 1972 under the name of massive sinus histiocytosis with lymphadenopathy [1, 2].

It is a rare condition, with fewer than 1,000 cases reported in the literature; most publications refer only to a limited number of cases [3–5].

Its cause is unknown; factors of genetic susceptibility have been suggested, given the often early age of diagnosis and the overrepresentation of subjects of black African origin, as well as the description of familial forms (Faisalabad syndrome) [6]. The hypothesis of a viral origin has been raised, but to date, there is insufficient evidence to reach a conclusion [1].

The most classic clinical aspect is the rapid appearance of cervical lymph nodes; other lymph node territories may be affected at diagnosis. In reality, the clinical picture is more multifaceted. Extranodal involvement now accounts for most of the reported cases [7]. In biology, there is a biological inflammatory syndrome in 2/3 of the cases; stigmata are sometimes observed, sometimes with a positive Coombs test [1].

This is a classic case from an epidemiological and clinical point of view. We report the case of an 8-year-old girl of Senegalese origin with a rapidly progressive cervical lymph node mass and a biological inflammatory syndrome.

However, the rarity of RDD means that it is often overlooked by physicians, especially in tropical settings, leading to diagnostic errors and delays [8]. In Senegal, Kane et al. [9] reported 3 cases of RDD, 2 of which were initially diagnosed and treated as lymphoma and tuberculosis, respectively, on the basis of suggestive lymph node cytology, without histology.

The main differential diagnoses were lymphoma, other histiocytoses, lymph node metastases of ENT cancers (cavum++) and paraganglioma, tuberculosis, and persistent lymphadenopathy in the course of HIV, especially in tropical settings. The epidemiological and clinical context, including age, the course of the disease, and the presence or absence of general signs, should rapidly guide the diagnosis. In all cases, histology is always decisive, with the characteristic appearance of massive lymphocytic infiltration and images of emperipolesis [1].

Currently, there are no codified therapeutic strategies. In most cases, no treatment is given, as the disease often progresses spontaneously. The most commonly used treatment is corticosteroid therapy [1]. The main role of corticosteroid therapy in the treatment of RDD is its anti-inflammatory and anti-immune action.

Some authors have reported the relative efficacy of the combination of 6-mercaptopurine and methotrexate in certain aggressive forms [9].

Azathioprine has also been reported to be effective, particularly in patients with RDD associated with a systemic disease sensitive to this treatment [10]. The efficacy of monoclonal antibodies, in particular, rituximab and siltuximab, has been reported in some cases [11, 12]. High-dose interferon has shown an objective response in one in 2 treated patients [9]. Analysis of these data suggests that immunosuppressive treatment is more often effective than cytotoxic treatment.

In our case, we decided on corticosteroid therapy due to the rapidly progressive and voluminous nature of the cervical mass and the risk of compression. This treatment was effective, with the involution of the tumor syndrome in 1 month and no recurrence after 1 year.

## 4. Conclusion

RDD is a rare benign condition of unknown cause. The lack of awareness of the condition by physicians often leads to misdiagnosis, especially in tropical settings. Histology is, therefore, an essential part of the diagnosis. Corticosteroid therapy is an effective and accessible treatment, which often makes it the first-line treatment. An updated international registry is needed to increase knowledge of this disease. Codified therapeutic strategies should be proposed to harmonize practices.

## Figures and Tables

**Table 1:** Laboratory values of CBC and inflammatory parameters (at diagnosis and after 1 month of corticotherapy).

Biological parameters	At diagnosis	After 1 month of treatment
Complete blood count (CBC)	WBC = 5.7 G/L	WBC = 8/9 G/L
	Hgb = 9.8 g/dl	Hgb = 11.8 g/dl
	Reticulocytes = 45.6 G/L	
	Platelets = 547 G/L	Platelets = 463 G/L

Inflammatory parameters	C-reactive protein (CRP) = 48 mg/l	CRP < 6 mg/l

## Data Availability

The biochemical and laboratory data used to support the findings of this study are available from the corresponding author upon request.
